# Variability, repeatability and test-retest reliability of equine flash visual evoked potentials (FVEPs)

**DOI:** 10.1186/s12917-020-02463-8

**Published:** 2020-07-29

**Authors:** L. Ström, J. Bröjer, B. Ekesten

**Affiliations:** Department of Clinical Sciences, Swedish University of Agricultural Sciences, PO Box 7054, SE-750 07 Uppsala, Sweden

**Keywords:** Visual evoked potentials, VEP, FVEP, Vision, Horse, Variability, Test-retest, Repeatability

## Abstract

**Background:**

Visual evoked potentials (VEPs) are electrical potentials generated by neurons in the central nervous system in response to visual stimuli. A series of positive and negative wavelets in response to flash-stimuli (flash-VEP; FVEP) or reversing, iso-luminant patterns (pattern-VEP; PVEP) are recorded. Pathological conditions affecting the post-retinal pathways can alter overall waveform morphology, and also affect wavelet peak times and amplitudes. FVEPs have recently been described in horses, but more data on the variability within and between subjects is required, to adequately interpret results from clinical equine patients. Therefore, the purpose of this study was to describe the variability, repeatability and test-retest reliability of equine FVEPs in normal, adult horses.

**Results:**

Equine FVEPs were recorded from one randomly selected eye in 17 horses, from both eyes in eight of these horses, and also at two separate recording sessions in six horses. N1, P2, N2 and P4 wavelets were present in 100% of the recordings in all horses, while P1, N2a, P3 and P5 were only present in some recordings. Coefficients of variation (CVs) were low for P2, N2 and P4 peak times, but higher for all amplitudes. There were no statistically significant differences comparing peak times and amplitudes between eyes or between sessions. Coefficients of repeatability (CRs) are reported for P2, N2 and P4 peak times between eyes (P2; 5 ms, N2; 18 ms, P4; 18 ms) and also between sessions (P2; 5 ms, N2; 16 ms, P4; 39 ms). Intraclass correlation coefficients (ICCs), as an estimate of test-retest reliability, was assessed to be fair to excellent for most parameters.

**Conclusions:**

This study provides important data on variability, repeatability and test-retest reliability of FVEPs in normal, adult horses. We conclude that P2, N2 and P4 peak times should be included in the evaluation of equine FVEPs. The large inherent variability of FVEP amplitudes is likely to make them less suitable and useful for establishing a diagnosis on their own in most clinical patients, but they may occasionally provide support to a clinical diagnosis.

## Background

Visual evoked potentials (VEPs) are electrical potentials generated by neurons in the central nervous system in response to visual stimuli. These potentials in response to flash-stimuli (flash-VEP;FVEP) or reversing, iso-luminant patterns (pattern-VEP;PVEP), are recorded by electrodes placed on the scalp over the visual cortex. Several pathological conditions affecting the post-retinal pathways can alter overall morphology of the recorded waveform and also affect wavelet peak times and amplitudes [1].

In human medicine, recording of VEPs has many clinical indications including evaluation of patients with optic neuropathies, lesions compressing post-retinal pathways, Alzheimer’s disease, glaucoma and central visual impairment (CVI) of different etiologies [2–7]. It is well known that the appearance of FVEPs is variable [1, 8, 9] and it has been shown that PVEPs have lower variability in peak times, amplitudes and waveform morphology, both within and between subjects [1, 10–12]. Therefore, PVEPs are most often preferred in clinical human medicine. However, recording of PVEPs require cooperation by the patient (fixation and focus on the pattern) and FVEPs are therefore usually still the test of choice in infants, as well as in uncooperative, sedated and comatose patients [1].

**Fig. 1:**
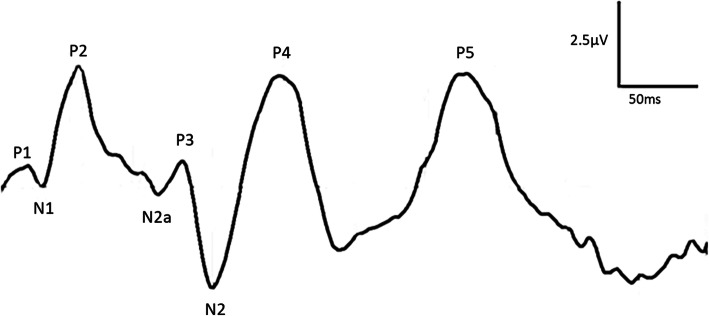
The equine FVEP. A series of positive and negative wavelets can be observed in the equine FVEP

In animals that cannot describe subjective symptoms, it is difficult to evaluate visual impairment. Vision is often severely reduced before the clinician can make a definitive diagnosis of disturbed vision. The FVEP has been used as an adjunct and objective test in the clinical work-up of animal patients [13–15]. Although FVEPs have been described in normal horses (Fig. 1) [16, 17], more data on the variability within and between subjects and evaluation of test-retest reliability is required, to adequately interpret results from clinical equine patients. Therefore, the purpose of this study was to describe the variability, repeatability and test-retest reliability of FVEPs recorded from healthy horses.

## Results

Intra- and inter-individual variability for the three replicates for each parameter are reported in Table 1. N1, P2, N2 and P4 peaks were present in 100% of recordings in all horses, while the remaining peaks (P1, N2a, P3 and P5) were only present in some recordings (Table 1). In general, intra- and inter-individual coefficient of variation (CV) values were lower for peak times compared to amplitudes (Table 1).

**Table 1:** Evaluation of three FVEP triplicates from one randomly selected eye in 17 horses

Parameter	No / 51 recordings	% of recordings	Mean	SD_intra-individual_	SD_inter-individual_	CV_intra-individual_	CV_inter-individual_
P1 (ms)	20	39%	15	3.4	2.3	23%	15%
N1 (ms)	51	100%	25	4.1	4.2	16%	17%
P2 (ms)	51	100%	54	3.3	3.6	6%	7%
N2a (ms)	29	57%	99	7.9	13.5	8%	14%
P3 (ms)	29	57%	115	11.2	7.2	10%	6%
N2 (ms)	51	100%	136	9.8	7.0	7%	5%
P4 (ms)	51	100%	213	14.9	23.8	7%	11%
P5 (ms)	14	27%	345	12.0	23.6	3%	7%
N1P2 (μV)	51	100%	4.1	0.8	2.6	19%	64%
P2N2 (μV)	51	100%	7.1	1.6	3.2	22%	45%
N2aP3 (μV)	29	57%	1.6	0.9	0.8	57%	49%
N2P4 (μV)	51	100%	7.7	2.3	2.8	30%	37%

FVEP triplicates from one randomly selected eye in each of 17 horses were evaluated in a nested mixed-model with random effects. Some wavelets were detected in all recordings, whereas others only in some replicates. Mean, intra-individual and inter-individual SD (SD_intra-individual_, SD_inter-individual_), as well as intra-individual and inter-individual coefficients of variation (CV_intra-individual_ and CV_inter-individual_ respectively) are reported for each parameter

Only wavelets present in all recordings were evaluated in the subsequent analyses. Table 2 show the descriptive data for the averaged measurement for each of these parameters in all horses.

**Table 2:** Descriptive FVEP data for measurements from all 17 horses

Parameter	Mean	SD	Range
P2 (ms)	54	4.0	46–59
N2 (ms)	136	9.0	117–152
P4 (ms)	213	25.3	170–271
N1P2 (μV)	4.1	2.7	1.6–10.2
P2N2 (μV)	7.1	3.3	3.8–13.8
N2P4 (μV)	7.7	3.1	3.7–13.9

Mean ± SD and range for measurements for each parameter from one randomly selected eye in 17 horses

**Fig. 2:**
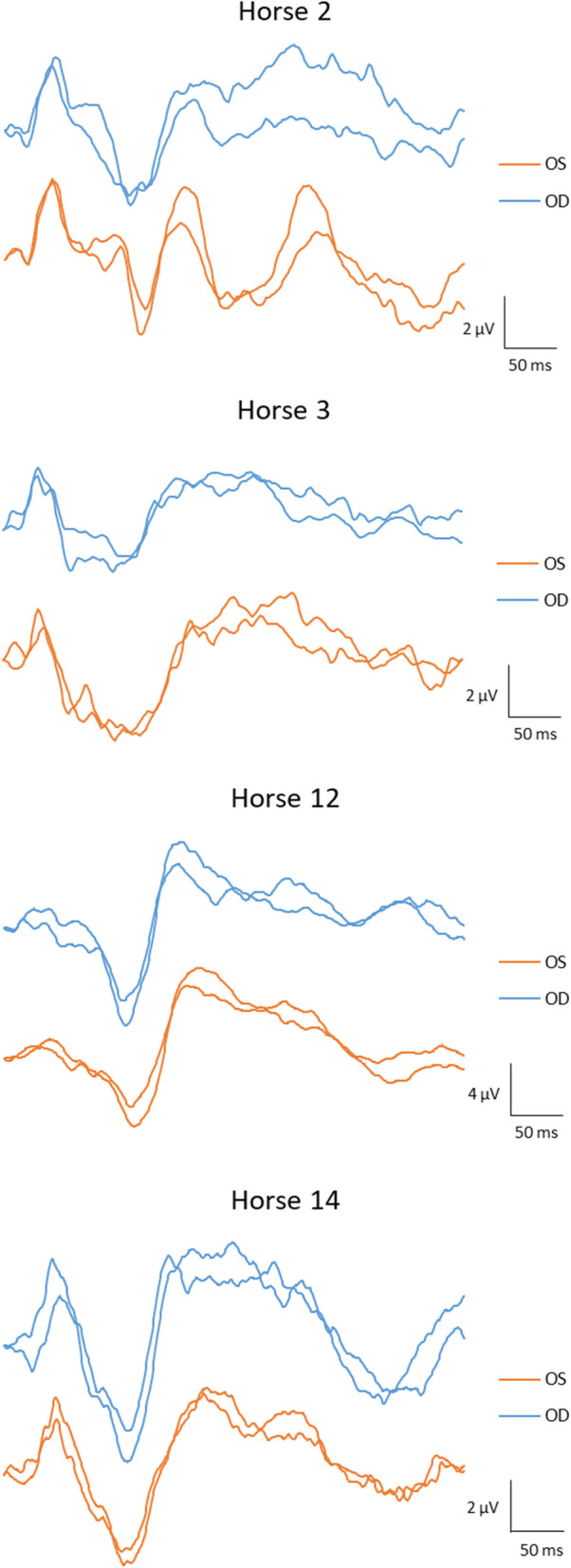
FVEP tracings from left and right eyes. Two tracings each from left and right eyes (OS and OD) recorded at the same session in four horses (horses 2–3, 12 and 14)

The FVEP waveform was similar between eyes (Fig. 2). There were no statistically significant differences between measurements from right and left eyes (*p* > 0.33) (Table 3). Coefficients of variation between right and left eyes were low for peak times but higher for amplitudes (Table 3). The computed CR between eyes for each parameter are reported in Table 3.

**Table 3:** Comparison of FVEP measurements from left and right eyes

	OD		OS				
Parameter	Mean	SD	Mean	SD	***p***-value	CV	CR
P2 (ms)	52	5.7	52	6.0	0.56	3%	5
N2 (ms)	138	8.1	134	10.8	0.33	5%	18
P4 (ms)	200	12.4	197	17.7	0.40	3%	18
N1P2 (μV)	2.5	0.9	2.5	1.3	0.99	28%	2.0
P2N2 (μV)	5.2	1.5	5.5	1.4	0.42	11%	1.7
N2P4 (μV)	5.9	2.6	6.3	2.7	0.43	14%	2.3

Results from recordings from left and right eyes (OS and OD, respectively) obtained from eight horses during the same recording session. Mean ± SD for left and right eyes. A paired t-test was used to evaluate results from left and right eyes, *p*-values< 0.05 were considered statistically significant. Coefficients of variation (CV) and coefficients of repeatability (CR) are reported

**Fig. 3:**
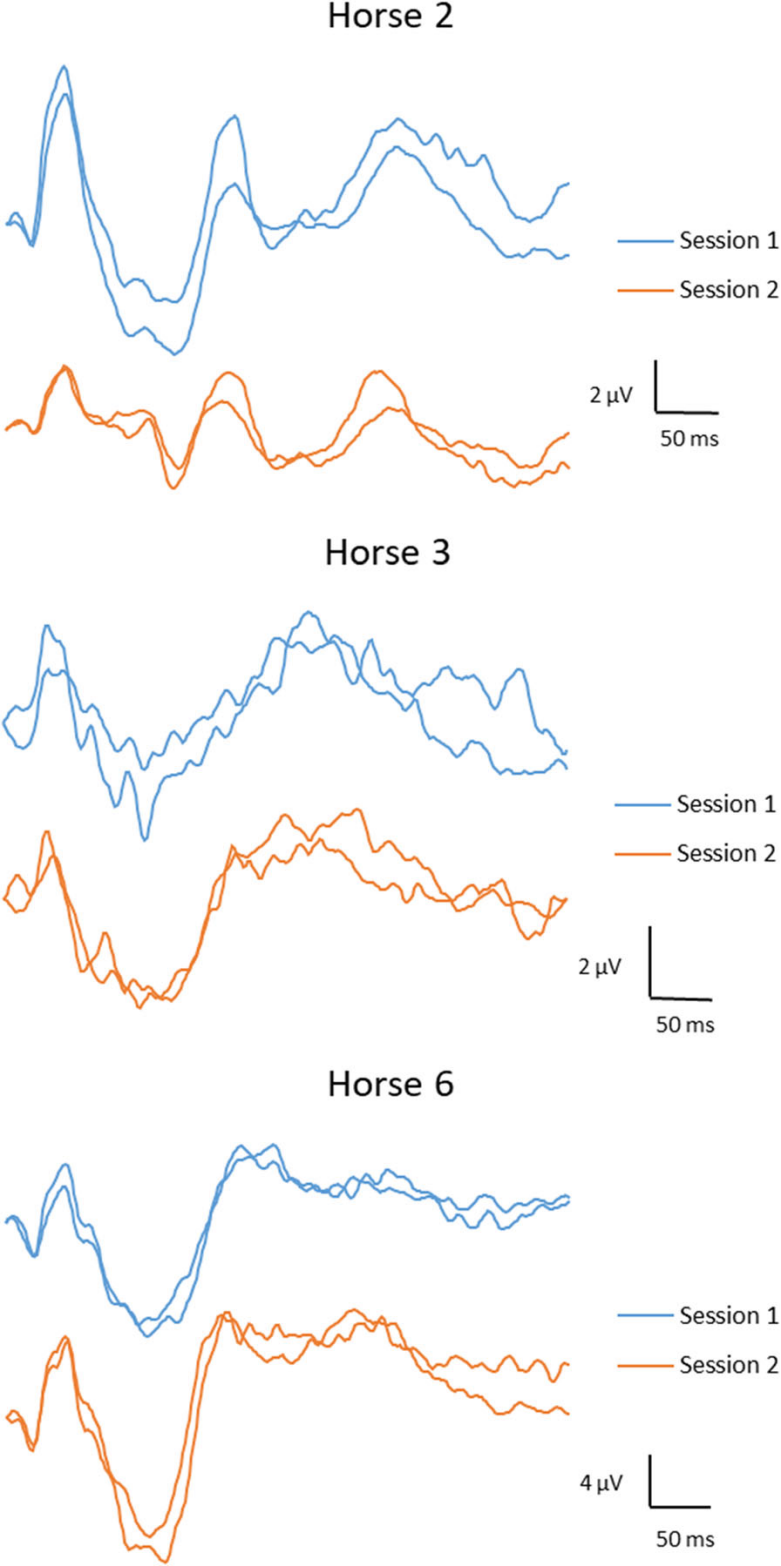
FVEP tracings from separate sessions. Two representative tracings from each of two separate recording sessions in three horses (horses 2, 3 and 6)

**Fig. 4:**
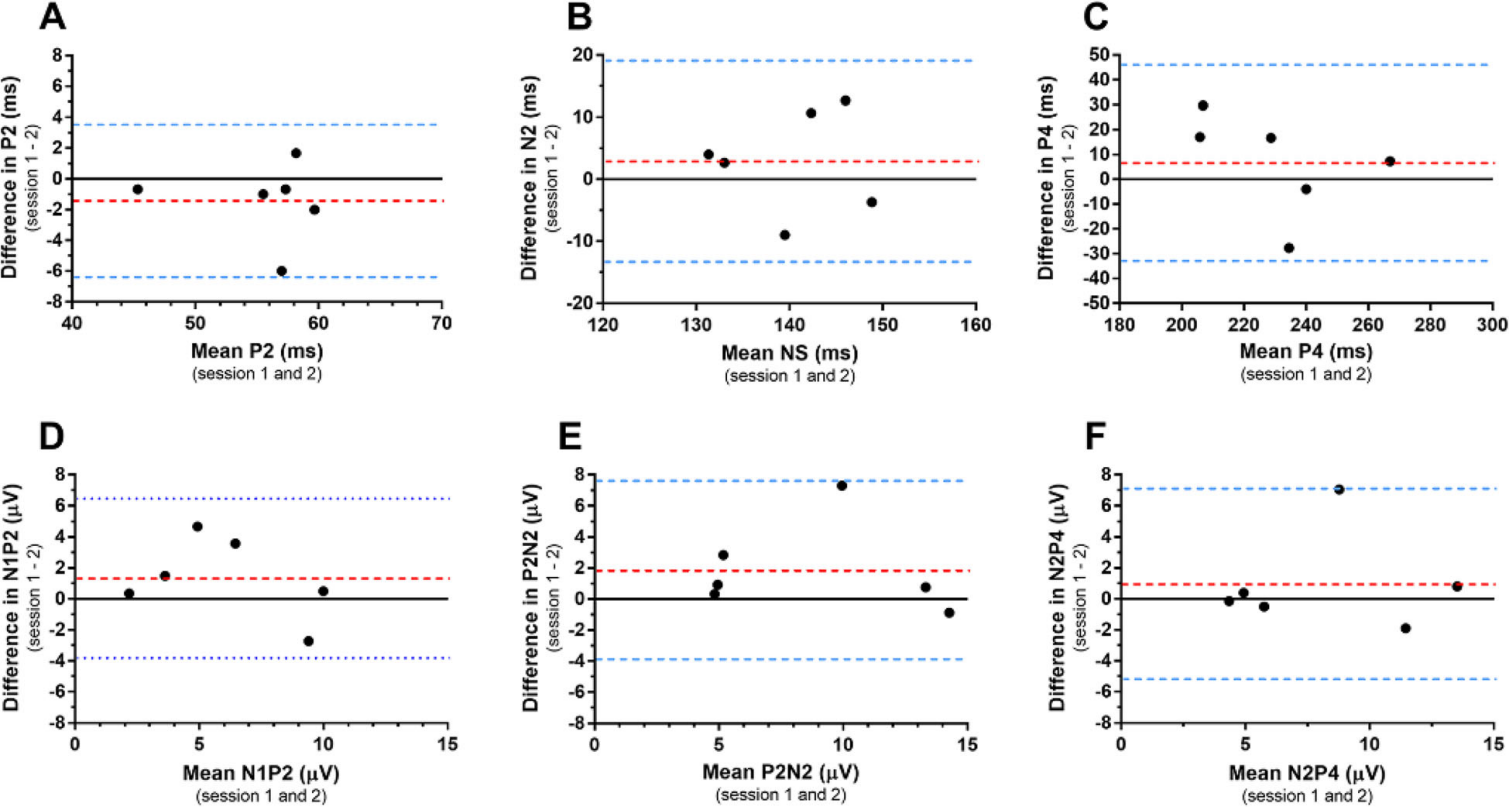
Bland-Altman plots to compare measurements between sessions. Bland-Altman plots with 95% limits of agreement for the mean difference (six horses). The mean measurement from the first and second recording session (x-axis) was plotted against the difference between the two measurements (y-axis). The mean difference was the estimated bias (dotted red lines). Limits of agreement are marked as blue dotted lines

FVEP waveforms were similar between recording sessions. Individual waveforms from two different recording sessions in three horses are displayed in Fig. 3. Bland-Altman plots including 95% LOA for P2, N2, P4 peak times and N1P2, P2N2, N2P4 amplitudes are shown in Fig. 4. The plot illustrates the agreement in measurement between session 1 and 2. There were no statistically significant differences between first and second measurements for any of the parameters (Table 4). Coefficient of variations were low for peak times, but higher for amplitudes (Table 4). The computed CR and intraclass correlation coefficients (ICCs) for each parameter are reported in Table 4.

**Table 4:** Comparison of FVEP measurements from two sessions

	Session 1		Session 2					
Parameter	Mean	SD	Mean	SD	p-value	CV	CR	ICC
P2 (ms)	55	5.2	56	5.5	0.65	3%	5	87%
N2 (ms)	142	8.3	139	7.9	0.55	4%	16	43%
P4 (ms)	234	20.4	227	28.8	0.66	6%	39	63%
N1P2 (μV)	6.8	2.9	5.4	3.8	0.52	30%	5.1	67%
P2N2 (μV)	9.7	4.4	7.8	4.8	0.49	24%	5.7	78%
N2P4 (μV)	8.6	4.2	7.7	4.0	0.69	27%	6.1	66%

Results from unilateral FVEPs from six horses at two separate recording sessions months apart. Mean ± SD for first and second sessions. A paired t-test was used to compare results from the two sessions, *p*-values< 0.05 were considered statistically significant. Coefficients of variation (CV), coefficients of repeatability (CR) and intraclass correlation coefficients (ICC) are reported

## Discussion

We have examined the variability, repeatability and test-retest reliability of FVEPs in normal, adult horses to evaluate if this method may be a suitable, additional diagnostic tool to use in the clinical work-up of equine patients with ophthalmological and/or neurological disease. Before a new method can be used in clinical practice, its strengths and weaknesses have to be assessed, in order to correctly interpret results from clinical patients and be able to make adequate clinical decisions.

The FVEP waveform recorded in all 17 horses in this study consisted of a series of positive and negative wavelets (P1, N1, P2, N2a, P3, N2, P4 and P5), as previously reported [16, 17]. However, some wavelets were only present in a limited number of recordings in all horses. This is similar to results from several human studies. Gastaut & Regis [8] showed that only about 20% of recordings included all wavelets in the human FVEP (peaks I-VI according to their nomenclature), but wavelets IV (around 80 ms) and V (around 130 ms) were present in practically all recordings and in all subjects. In the ISCEV standard for human FVEPs, the waveform is defined as a series of positive and negative wavelets (N1, P1, N2, P2, N3 and P3). Of these wavelets, N2 (around 90 ms) and P2 (around 120 ms) are described as the most robust components [1]. In our study on the equine FVEP, wavelets N1, P2, N2 and P4 were present in all recordings in all 17 horses. Both the intra- and inter-individual coefficients of variation were low (5–11%) for the peak times of P2, N2 and P4. Although present in all recordings, the N1 peak time was shown to be more variable (up to 17%), probably because this low-amplitude wavelet is occasionally difficult to localize and measure precisely. Our results support that the P2, N2 and P4 peak times are most robust, and should be included in the evaluation of equine clinical patients. Although not present in all recordings, the N2a, P3 and P5 wavelets may still provide valuable clues regarding transmission and processing of visual stimuli. Hence, they may be useful for evaluation of certain conditions, or be valuable for our understanding of processing of visual input, but this warrants further studies.

Our results show that there is a substantial intra- and inter-individual variability in amplitudes in the equine FVEP. A substantial variability in amplitudes has also been shown in studies on the human FVEP [8, 9]. In our study, the coefficients of variation for the amplitudes present in all recordings in all horses (N1P2, P2N2 and N2P4), were up to 30% within horse and more than twice that (up to 64%) between horses. Hence, the variation for these amplitude parameters was considerably higher than that for peak times. Based on the results of our study, a wide range of amplitudes must be considered normal in the FVEP recorded from sedated horses. Therefore, the mere presence of a wavelet and its peak time may often be sufficiently informative, as very few patients are likely to have amplitudes that fall outside of the normal range and only severe abnormalities will cause sufficiently abnormal amplitudes.

The waveforms obtained from left and right eyes during the same recording session were similar. The variability in peak times between eyes in the same horse was low, only 7% or less. The variation has also been described to be quite similar between eyes within the same subject in humans [1]. This low inter-ocular variation enhances evaluation of clinical patients with suspected unilateral dysfunction, when one eye can serve as a control eye. The coefficients of repeatability reported in our study represent the range within which the absolute difference between two measurements on the same subject should fall with a 95% probability. Larger differences, outside the range set by the CR values (P2; 5 ms, N2; 18 ms, P4; 18 ms), are likely to indicate abnormal function. Again, amplitudes were shown to be more variable between eyes. However, the P2N2 and N2P4 amplitudes may provide important information, with CR values at 1.7 μV and 2.3 μV, respectively.

The waveforms obtained at separate recording sessions appeared quite similar. The coefficients of variation for peak times between sessions were low (3–6%) but higher for amplitudes (24–30%), which is similar to the variability shown between eyes within the same session. Bland-Altman plots with 95% limits of agreement were used to graphically examine the agreement between two measurements from separate recording sessions. The plots show that the mean difference between sessions is low for the P2 peak time, but higher for the N2 and P4 peak times. The mean differences were similar across all amplitude parameters. The coefficients of repeatability were computed to quantify the absolute repeatability in the same unit as the parameter with a probability of 95%. Based on our results, differences in peak times between recording sessions falling outside the reported CR values (P2; 5 ms, N2; 16 ms, P4; 39 ms), are likely to indicate either an improvement or deterioration of a condition. For the amplitudes, the CR values are higher, again supporting the conclusion that differences in amplitudes between sessions only rarely will provide reliable information regarding the progression of a disease or effect of a treatment. The ICC is a widely used reliability index in test-retest analyses [18]. In our study on the equine FVEP, we found that the ICC values ranged from fair (N2 peak time) to excellent (P2 peak time and P2N2 amplitude) according to the grading system proposed by Cicchetti [19].

Some of the variability in our data is due to difficulties in establishing the peak or trough of a specific wavelet precisely. Large amplitude, pointed wavelets are generally easier to pinpoint compared to low-amplitude, or more rounded or elongated wavelets. N1 was usually a low-amplitude wavelet, where the trough sometimes was difficult to localize precisely. P2, on the other hand, was most often a distinct peak that was easy to discriminate. Thus, it is not surprising that the P2 peak time showed least variability and highest repeatability and reliability. Although the N2-complex always was easy to identify, this complex was sometimes wide with a flat bottom (not a distinct trough), and sometimes also included N2a and P3. Therefore, the exact position of N2 was occasionally difficult to determine. P4 was also most often easily discriminated, but in some horses this wavelet was wide without a distinct peak (for example horse 3 in Fig. 2), which made precise marking difficult. In spite of differences in the precise localization of some wavelets, coefficients of variation were low for P2, N2 and P4 peak times. The coefficients of repeatability, which represent the absolute difference between measurements (in the same unit as the parameter), show higher values for both N2 and P4 peak times compared to P2 peak time, which altogether is not surprising.

The difficulties in the precise localization of wavelets certainly affected the amplitude measurements for the same reasons mentioned above, although probably not as much as peak times according to our subjective assessment. The large variability shown for amplitudes is more likely due to other factors, such as variation in the level of sedation, muscle and movement artifacts, the temperament of the horse and its responses to external disturbances in the clinical environment. The variation between sessions may also be attributed to minor differences in electrode positions between sessions.

Andersson et al. [9] evaluated the test-retest properties of the human FVEP in 15 awake, normal subjects at three separate recording sessions. They found that precise marking of wavelets was sometimes difficult, due to split peaks and highly variable waveforms within and between individuals. Wide inter-individual ranges for both N2 and P2 peak times and amplitudes (the parameters they evaluated) were described, and a high intra-individual variability over time was reported. Specifically, they concluded that due to the large variability, the FVEP is unreliable as a tool for detecting increased intracranial pressure which had previously been suggested by other authors. Therefore, they advised caution when interpreting changes in FVEPs in clinical work. It is not possible to make direct comparisons between human and equine FVEPs, because of differences in the overall waveform between species, different electrode positions due to anatomical dissimilarities, and the fact that our horses were sedated and their human subjects fully awake. All these differences may certainly have an impact on the variability. However, some comparisons may still be relevant. We saw some individual variation in waveforms between horses (Fig. 2 and Fig. 3), which is similar to what was described by Andersson et al. [9] in their human subjects. As in our study, Andersson et al. [9] report that deciding on the exact position of a peak or trough of a wavelet was sometimes difficult. However, our impression is that the wavelets were somewhat easier to discriminate in the equine FVEPs compared to the human FVEPs. Also, split peaks were not as prominent and frequent as described by Andersson et al. [9] in some human subjects. The range is narrower for P2 and N2 peak times in the equine FVEP (Table 2), compared to more than 50-ms-intervals considered to represent the normal ranges for N2 and P2 in the human study.

A limitation of our study is the small sample size, which is due to limited access to horses available for the study. Reported values for reliability and repeatability should therefore be interpreted with some caution. In addition, further studies are needed, to evaluate equine FVEPs in horses with diseases in visual pathways, causing visual impairment. Although we have provided CR values between eyes and sessions in equine FVEPs, their clinical significance warrants further studies. Differences lower than the reported CR values (between eyes and sessions) can still be of importance, because mild dysfunction of the visual pathways may still be present. Therefore, results from FVEP testing should always be put into context with other findings and results from additional tests obtained during work-up of a patient, including for example ophthalmic and neuro-ophthalmologic examinations, obstacle course testing and diagnostic imaging.

## Conclusions

In summary, this study provides important data on variability, repeatability and test-retest reliability of FVEPs in normal, adult horses. Based on our data, we conclude that P2, N2 and P4 peak times should be included in the evaluation of equine FVEPs. The large inherent variability of FVEP amplitudes is likely to make them less suitable and useful in most clinical patients, but they may occasionally provide support to a clinical diagnosis. Although all laboratories performing electrophysiological examinations are recommended to produce and use own normative data when evaluating clinical patients, this study provide information on the magnitude of the variability and repeatability that can be expected in the normal horse. To assess the full potential of FVEPs as a diagnostic aid in equine clinical practice, further evaluation of results from horses with visual impairment of different etiologies is warranted.

## Methods

### Horses

Seventeen healthy adult Standardbreds (3–19 years old, mean ± standard deviation (SD); 10 ± 5.7 years, 4 geldings, 2 stallions and 11 mares), without any signs of ocular, visual or neurological disease at physical and ophthalmic examination, including obstacle course testing, were included in the study. Horses 1–10 and 1–17 were also included in two previous studies [16, 17]. The study was approved by the Regional Ethical Committee (Uppsala Djurförsöksetiska nämnd, Sweden, C254/10 and C39/12). The experiments were carried out following national and institutional guidelines for care and use of animals in research. All horses were owned by the Swedish University of Agricultural Sciences and they were alive and healthy after the experiments.

### FVEPs

Light-adapted FVEPs were recorded according to the protocol described by Ström & Ekesten [17]. All recordings were performed in a clinical examination room at the Horse Clinic at the Swedish University of Agricultural Sciences. Horses were sedated with an intravenous bolus injection of detomidine, 0.01 mg/kg (Domosedan vet., 10 mg/ml, Orion Pharma Animal Health, Sollentuna, Sweden) followed by maintenance of sedation throughout the recording sessions using a continuous intravenous infusion of 2% detomidine in physiologic saline solution (Natriumklorid, 9 mg/ml, Fresenius Kabi, Uppsala, Sweden), to keep the horse resting its head steadily on a padded headstand. Pupils were dilated (Mydriacyl, 0.5%, Novartis, Stockholm, Sweden), akinesia and analgesia of the eyelids and electrode positions were performed to avoid muscle artifacts (Carbocain, 20 mg/ml, AstraZeneca, Södertälje, Sweden), and topical corneal anesthesia was applied (Tetrakain, 1%, Bausch & Lomb Nordic AB, Stockholm, Sweden). The room was kept dark during recordings to avoid stray light. The fellow eye was covered by a black, opaque plastic eye shield, and cotton wads were used in the ears to reduce auditory stimuli.

Cork-screw electrodes were used (Stainless Steel Disposabel Corkscrew Electrode, Cephalon A/S, Nörresundby, Denmark). The active electrode was placed at P_z-45_, the ground and reference electrode were placed on the forehead in the midline according to Ström & Ekesten [16]. Electrode impedance was kept below 5 kΩ. The flash stimulus was elicited by a handheld dome-shaped LED-photostimulator with a background light intensity of 25 cd/m^2^, a flash intensity of 3 cd/m^2^/s and a flash duration of 5 ms. The stimuli were presented at a frequency of 1.09 Hz. The recording window was 500 ms. Responses were amplified (amplifier set to × 10^4^), averaged (100–144 averages), band-pass filtered (1–100 Hz), stored and analyzed using the An-Vision RETI-port (An-Vision, Hennigsdorf, Germany) and a laptop computer. Light-adapted ERGs were recorded simultaneously to assure that a normal response was generated by the retina.

The same examiner (LS) performed all recordings. Equine FVEPs were recorded from one randomly selected eye in 17 horses (left eye; OS, right eye; OD), in both eyes (OU) in eight horses and also at two separate recording sessions (2–11 months apart, median 7.5 months) in six horses (Table 5). According to the human standard protocol of the International Society for Clinical Electrophysiology of Vision (ISCEV) [1], a minimum of two reproducible recordings should be performed in clinical VEP testing. Obtaining several reproducible replicates is described to be even more important in pediatric subjects, to assure that the response recorded is a reliable signal and not an artifact due to poor cooperation. Therefore, from the horses in our study (which may be compared to slightly uncooperative pediatric subjects), we chose to obtain three replicates from each horse and eye stimulated.

**Table 5:** Description of horses and FVEP recordings performed in the study

Horse	Age (years)	Gender	No of sessions	Time between sessions (months)	Eye (s) examined
1	5	M	2	7	OS
2	10	M	2	6	OU
3	10	F	2	7	OD
4	16	F	2	11	OU
5	16	F	2	11	OD
6	19	F	2	5	OD
7	3	F	1	–	OU
8	4	F	1	–	OU
9	5	M	1	–	OU
10	17	F	1	–	OS
11	3	F	1	–	OU
12	3	F	1	–	OS
13	6	M	1	–	OD
14	7	F	1	–	OD
15	12	F	1	–	OU
16	14	F	1	–	OU
17	16	M	1	–	OS

Age at first examination. *M* male, *F* female, *OS* left eye, *OD* right eye, *OU* both eyes

### Data analysis

One author (LS) performed all markings of tracings to avoid investigator dependent variability. Wavelets were identified and named in accordance with the previously described FVEP waveform in horses (Fig. 1) [16]. Peak times and amplitudes were evaluated according to established guidelines [16, 20]. Blinded marking of each identified parameter in all three replicates was performed. Data were assessed for normality by visual examination of residual plots prior to analysis. Parameters were tested for homogeneity of variance using Levene’s test. Statistical analysis was performed using a statistical software (JMP® Pro 14.0.0). The level of statistical significance was defined at *p* < 0.05.

To assess the intra- and inter-variability of the three replicates for each parameter (peak times and amplitudes), a nested mixed-model with random effects was used. Only peak times and amplitudes containing 100% recordings were used for the remainder of the analyses. The average of the three replicates were calculated and used as one measurement for each parameter, when comparing results between eyes (eight horses) and between sessions (six horses). Parameters were compared between left and right eyes, and between sessions for the same eye using a paired t-test. The repeatability for measured parameters between eyes in the same horse during the same recording session, as well as the repeatability for the same parameters for the same eye on different sessions were determined by the coefficient of variation (CV) and coefficient of repeatability (CR), whereas ICC was used to evaluate test-retest reliability between sessions.

Coefficient of variation is a measure of the relative variability, and was calculated using the SD within and between subjects obtained from one way ANOVA (within) or a nested mixed model (within and between) [21]. Coefficient of repeatability shows the absolute difference in peak time and amplitudes between measurements, and was computed according to the formula:

$$ 1.96\times \sqrt{2}\times \mathrm{SD}\;\mathrm{within}\ \mathrm{subject} $$

based on results from an ANOVA table [18]. Intraclass correlation coefficients (ICCs) were calculated to assess and quantify test-retest reliability for measurements between sessions. The ICC was calculated from an ANOVA table according to the formula

$$ \mathrm{ICC}={{\mathrm{S}}_{\mathrm{b}}}^2/\left({{\mathrm{S}}_{\mathrm{b}}}^2+{{\mathrm{S}}_{\mathrm{w}}}^2\right) $$

where the S_w_^2^ is defined as the within-subjects variance and S_b_^2^ as the between-subjects variance [18]. There is no universally agreed level for ICC values, but the often quoted guidelines by Cicchetti [19] were used for interpretation of ICC reliability: poor: < 0.40, fair: 0.40–0.59, good: 0.60–0.74, excellent: 0.75–1.00.

Bland-Altman plots including 95% upper and lower limits of agreement were constructed based on measurements from recordings at two separate sessions [22, 23]. The average measurement from the two recording sessions for each subject was plotted against the difference between measurements to assess repeatability between sessions.

## Data Availability

The datasets used and/or analyzed during the current study are available from the corresponding author on reasonable request.

## Abbreviations

CI: Confidence interval
CR: Coefficient of repeatability
CV: Coefficient of variation
FVEP: Flash visual evoked potential
ICC: Intraclass correlation coefficient
LOA: Limits of agreement
OD: Right eye
OS: Left eye
OU: Both eyes
PVEP: Pattern visual evoked potential
VEP: Visual evoked potential
